# Effect of Arginine Infusion on Ghrelin Secretion in Growth Hormone Sufficient and GH Deficient Children

**DOI:** 10.5812/ijem.3826

**Published:** 2012-04-20

**Authors:** Flavia Prodam, Giulia Genoni, Simonetta Bellone, Silvia Longhi, Valentina Agarla, Gianni Bona, Giorgio Radetti

**Affiliations:** 1 Division of Pediatrics, Department of Medical Sciences, University of Piemonte Orientale, Novara, Italy; 2 Endocrinology, Department of Clinical and Experimental Medicine, University of Piemonte Orientale, Novara, Italy; 3 Department of Pediatrics, Regional Hospital of Bolzano, Bolzano, Italy

**Keywords:** Acylated Ghrelin, Growth Hormone, Growth Hormone Deficiency

## Abstract

**Background:**

The physiological link between ghrelin and growth hormone (GH) has not yet been fully clarified. Furthermore, the existence of a negative feedback mechanism between growth hormone–insulin-like growth factor (GH–IGF)-I axis and ghrelin and the influence of amino acids on ghrelin secretion in children remain matters of debate.

**Objectives:**

To understand the regulation of ghrelin secretion and clarify the relationship between ghrelin and GH secretion in GH-deficient (GHD) and GH-sufficient (GHS) children.

**Patients and Methods:**

Ten GHD (male/female [M/F], 6/4; age [mean ± SEM], 10.7 ± 0.9 years) and 10 GHS prepubertal children (M/F, 6/4; age [mean ± SEM], 10.3 ± 0.6 years), underwent an arginine (ARG) test (infusion, 0.5 g/kg, iv). Levels of GH, total ghrelin, and acylated ghrelin (AG) were assayed every 30 min from 0 to +120 min.

**Results:**

Peak GH values were lower in GHD subjects than in GHS subjects (P < 0.0001). The baseline levels, peak levels, or area under the curves (AUC) for total ghrelin and AG were similar between GHD and GHS children. ARG infusion was followed by a slight to significant decrease in total ghrelin levels, but not AG levels, both in GHD and GHS subjects with a nadir at +30 min. No correlation was seen between GH, total ghrelin, or AG response and ARG infusion.

**Conclusions:**

Total ghrelin and AG levels seemed unaffected by GH status in prepubertal children. ARG infusion was unable to blunt ghrelin secretion irrespective of GH status in childhood. Moreover, since ARG influences GH secretion via modulation of somatostatin release, ghrelin secretion seems to be partially refractory to somatostatin action.

## 1. Background

Ghrelin is a 28 amino acid peptide. Although predominantly produced by the stomach, it is also produced by several other tissues, including the pituitary and hypothalamus (1-3). It is the first discovered natural ligand of the orphan growth hormone (GH) secretagogues receptor type 1a (GHS-R1a) and induces a strong GH-releasing activity on activation (1, 4-6). Ghrelin circulates in the blood in 2 different forms. Acylated ghrelin (AG) contains an acyl group bound to the octanoyl group on a serine-3 residue of the molecule; acylation at serine-3 is essential for GHS-R1a activation (1, 5) Unacylated ghrelin (UAG) is the commonly present circulating form and it has a lot of physiological functions (7-9). Circulating ghrelin levels are mainly influenced by the nutritional and metabolic statuses of an individual, and the levels increase with fasting and energy restriction and decrease with food intake, glucose metabolism, and insulin secretion (10-12).

GH secretion from the pituitary gland is regulated by the coordinated action of GH-releasing hormone (GHRH) and somatostatin (SRIH), which, respectively, stimulate and inhibit the release of GH (13, 14). GH secretion is also influenced by metabolic and hormonal signals from other glands, including release of glucocorticoids, thyroid hormones, and sex steroids, which may act directly or via hypothalamic connections. Furthermore, GH regulates its own secretion by a feedback mechanism. Other peripheral mediators, such as insulin-like growth factor I (IGF-I), free fatty acids, glucose, and insulin, can act as a part of this feedback mechanism (15).

The GH-releasing effect of ghrelin is mediated by GHRH-secreting neurons at the hypothalamic level. Ghrelin and GHRH have synergic activities indicating that they act, at least partially, by different mechanisms of action (16, 17). Nevertheless, ghrelin needs GHRH activity to fully exert the GH-releasing effect. Since ghrelin mainly acts at the hypothalamic level, it requires the integrity of hypothalamic-pituitary connections (18, 19). Furthermore, ghrelin is an antagonist of SRIH at both the hypothalamic and pituitary levels (3, 20).

Despite the stimulatory effect of acute ghrelin administration on GH secretion, the physiological link between ghrelin and GH has not yet been clarified (21, 22). The following conflicting results have been obtained in clinical studies: (a) existence of a relationship between GH and circulating ghrelin levels (23), (b) absence of a relationship between GH and ghrelin levels (24), and (c) existence of a relationship between GH and ghrelin levels under specific conditions such as fasting or the time of day (25).

Furthermore, many studies have been conducted in short-statured children and adults to assess the existence of a negative feedback mechanism between the GH–IGF-I axis and ghrelin by using different GH provocative tests; however, the results remain controversial (26-30).

## 2. Objectives

We studied total ghrelin and AG secretion with respect to GH response in a classical GH-stimulation test in GH-deficient (GHD) and GH-sufficient (GHS) children. The aims of this study were to understand the regulation of ghrelin secretion and the relationship between ghrelin and GH secretion in a better way and to clarify the influence of amino acids on ghrelin secretion in children.

## 3. Patients and Methods

Between November 2008 and June 2010, 20 short-statured children, admitted to the Division of Pediatrics, University of Eastern Piedmont, Novara, Italy, were enrolled in this study. Informed parental consent and approval by the local ethical committee were obtained before beginning the study.

Inclusion criterion was a short stature, which was defined as height with a standard deviation (SD) score below -2 on Italian growth charts (31). Exclusion criterion was the presence of a known cause of short stature, such as hypothyroidism, chronic diseases, or celiac disease; small for gestational age children were excluded.

On the basis of cut-off levels of GH response to classical stimulation tests for the diagnosis of GH deficiency (peak GH level, < 10 ng/mL) in children (32), we classified 10 patients as GHD and 10 as GHS. The subjects underwent a complete clinical examination, and auxological parameters were evaluated according to Italian growth charts (31). Height and weight were, respectively, measured by using the Harpenden stadiometer and an electronic scale. Body mass index (BMI) was calculated as body weight divided by squared height (kg/m^2^), and BMI SD score was assessed using the least mean square method (LMS method) (31). Brain magnetic resonance imaging (MRI) was performed for all GHD children and the condition was classified as idiopathic GH deficiency. The median follow-up time for the assessment of growth velocity and other auxological parameters was 6 months.

All subjects underwent an arginine test (ARG) (infusion, 0.5 g/kg, iv, maximum 30 g, within 30 min) from 8:00 to 8:30 AM, after overnight fasting. An intravenous catheter was placed in the antecubital vein half an hour prior to the test. Blood was sampled at baseline and 30, 60, 90, and 120 min after ARG infusion. Serum GH and ghrelin level were measured at each test point (33), while IGF-I concentration was measured only at the baseline.

For serum preparations, GH and IGF-I were collected in tubes containing no anticoagulants while total ghrelin and AG were directly drawn into tubes containing potassium ethylenediaminetetraacetic acid (K_2_EDTA). AG plasma samples were centrifugated (15 min, 3000 rpm, 4°C), acidified with HCl (1 M), and pretreated with a protease inhibitor cocktail. The samples were stored at -80°C.

All samples were analyzed within 15 days from the day of collection.

Plasma GH, IGF-I, AG, and total ghrelin levels were measured using commercially available kits.

During provocative tests, GH levels were measured using a chemiluminescent immunoassay system (IMMULITE 2000, Siemens); serum IGF-I levels were measured using LIASION automated chemiluminescence analyzer, supplied by DiaSorin, with a measurement range of 3–1,500 ng/mL. To determine the immunoreactive ghrelin concentration, total ghrelin levels (pg/mL) in plasma were assayed in duplicate by using a commercially available radioimmunoassay (RIA) (Phoenix Pharmaceuticals, Mountain View, CA, USA) following the manufacturer’s recommendations. Intra- and inter-assay coefficients of variation (CV) were below 5.3% and 13.6%, respectively. Plasma AG levels (pg/mL) were measured in duplicate using a commercial RIA kit (LINCO Research Inc, Millipore, MO, USA) following the manufacturer’s recommendations. The antibody showed 100% cross-reactivity to human AG and < 0.1% cross-reactivity to unacylated human ghrelin. Intra- and inter-assay CV values were below 13.9% and 20.4%, respectively. Data were expressed as mean ± SEM. Area under the curves (AUC) was calculated using trapezoidal integration. A sample size of 10 individuals from each group was required to provide sufficient power (80%) to detect a difference of 50 pg/mL with SD of 40 pg/mL at a significance level of 95% between the mean ghrelin levels in the 2 groups (34). For continuous variables, the variation between the groups was compared using nonparametric Wilcoxon and Mann–Whitney U tests, as appropriate. A correlation analysis was performed using the Spearman’s correlation test. A p value of < 0.05 was considered statistically significant. All statistical analyses were performed using SPSS for Windows version 15.0 (SPSS INC; Chicago, IL, USA).

## 4. Results

**Figure 1:**
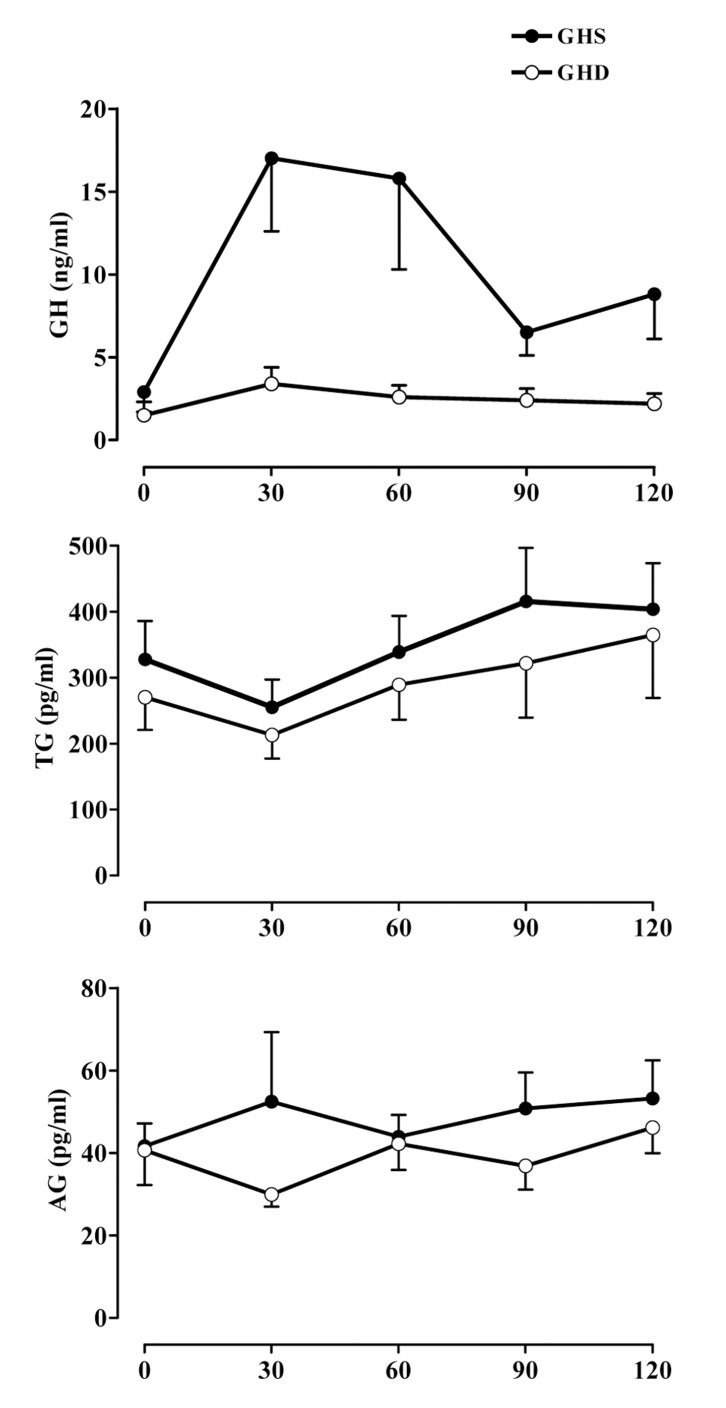
Mean ± SEM Values of Serum GH, Total Ghrelin, and Acylated Ghrelin (AG) during the Arginine Test in GH-Deficient (GHD) and GH-Sufficient (GHS) Children

Gender distribution and the mean age were similar between GHD and GHS children. All patients were prepubertal or in early puberty (Tanner pubertal stages I or II). All clinical and hormonal data are shown in Table 1. The peak GH values were significantly lower in GHD children (mean: 6.1 ng/mL, range: 3.9–8.4 ng/mL) than in GHS children (mean: 22.0 ng/mL, range: 12.1–62.3 ng/mL; P < 0.0001). IGF-I levels in GHD children (mean: 179.8 ng/mL, range: 27.0–399.0 ng/mL) were not statistically different from those in GHS children (mean: 250.9 ng/mL, range: 114.0–600.0 ng/mL) (Figure 1).

**Table 1:** Clinical and Hormonal Response to Arginine in GHD and GHS Children

		GHD b	GHS b
Case , No.	10	10
Gender (M/F) a	6/4	6/4
Age, y	10.7 ± 0.9	10.3 ± 0.6
Height, cm	128.6 ± 4.7	128.8 ± 3.6
Height, SDS a	-2.0 ± 0.1	-1.7 ± 0.1
Weight, kg	33.8 ± 4.5	24.7 ± 1.7
BMI a, kg/m ^2^	18.7 ± 2.2	15.2 ± 0.5
IGF-I level, ng/mL	179.8 ± 49.6	250.9 ± 50.4
IGF-I level, SDS a	-0.4 ± 0.2	0.2 ± 0.2
Baseline GH level, ng/mL	1.5 ± 0.8	2.9 ± 1.2
Peak GH level, ng/mL	6.1 ± 0.4 c	22.0 ± 4.7 c
AUC a GH, ng/mL×h	5.2 ± 0.8 c	22.4 ± 4.7 c
Baseline TG a level, pg/mL	270.3 ± 49.8	327.7 ± 58.3
Peak TG a level, pg/mL	392.9 ± 88.7	461.1 ± 69.7
AUC a TG a, pg/mL×h	554.7 ± 114.6	649.9 ± 109.7
Baseline AG a level, pg/mL	40.6 ± 8.4	41.6 ± 5.5
Peak AG a level, pg/mL	52.6 ± 7.0	73.6 ± 16.3
AUC a AG a, pg/mL×h	74.4 ± 7.7	90.4 ± 14.3

^a^Abbreviations: AG, Acylated ghrelin; AUC, Area under the curve; BMI, Body mass index; F, Female; H, Hour; M, Male; Sds, Standard deviation score; Tg, Total ghrelin; Ghs, Growth hormone sufficient; GHD, Growth hormone deficient

^b^Data are expressed as Mean ± SEM

^c^P < 0.0001

The baseline total ghrelin levels were similar between the 2 groups (GHS: mean, 327.7 pg/mL; range, 98.0–706.0 pg/mL; GHD: mean, 270.3 pg/mL; range, 50.2–519.6 pg/mL). Furthermore, the peak total ghrelin levels in the GHS children (mean: 461.1 pg/mL, range: 127.0–841.0 pg/mL) were not significantly different from those in GHD children (mean: 392.9 pg/mL; range: 153.0–1029.0 pg/mL). The AUC for total ghrelin levels was also similar for the 2 groups (GHS: mean, 649.9 pg/mL·h; range, 177.8–1365.9 pg/mL·h; GHD: mean, 554.7 pg/mL·h; range, 210.9–1359.6 pg/mL·h) (Figure 1).

The baseline AG levels were similar between the 2 groups (GHS: mean, 41.6 pg/mL; range, 27.9–74.4 pg/mL; GHD: mean, 40.6 pg/mL; range, 17.7–112.1 pg/mL). Furthermore, the peak AG levels in the GHS children (mean: 73.6 pg/mL, range: 25.5–201.1 pg/mL) were not significantly different from those in GHD children (mean: 52.6 pg/mL, range: 19.5–86.1 pg/mL). The AUC for AG levels was also similar for the 2 groups (GHS: mean, 90.4 pg/mL·h; range, 47.5–189.7 pg/mL·h; GHD: mean, 74.4 pg/mL·h; range, 38.9–105.4 pg/mL·h (Figure 1).

ARG infusion was followed by a slight to significant decrease in total ghrelin levels, but not AG levels both in GHD and GHS subjects, with a nadir at +30 min; however, the results were not statistically significant. No correlation was seen between GH response and total ghrelin and AG levels.

## 5. Discussion

This study evaluated the response of total ghrelin and AG level to a classical GH stimulation test in GHD and GHS children. Total ghrelin and AG levels were not modulated on acute ARG infusion irrespective of the GH status.

The lack of modulation of total ghrelin secretion is consistent with the findings of previous studies in which ARG alone or glucagon was administered in both children and adults with GHD and/or GHS (26, 35, 36). Interestingly, despite the remarkable effect of significant variation in circulating ghrelin levels on insulin, glucose, and GH levels, amino acids coupling was not observed. This finding is of particular interest taking into account that no effect was recorded after continuous infusion of ARG instead of acute bolus or oral loads, despite the effect of insulin and glucose levels associated with continuous infusion being stronger (35, 36). Moreover, since ARG affects GH secretion via modulation of SRIH release, ghrelin secretion seems to be partially refractory to SRIH action, which is also observed in adults (35). However, a recent study showed inhibition of ghrelin secretion after administration of GHRH and ARG in GHD and GHS adults. This decrease in ghrelin levels cannot be attributed to GH release induced by ARG (35, 36) and might be a result of an inhibitory effect exerted by GHRH (30). Oral clonidine administration has been reported to blunt ghrelin secretion in GHS children, although a delayed increase in ghrelin secretion was observed in GHD children (29). This effect of oral clonidine might be related to its direct gastric action since it is known to play a role in delaying gastric emptying, inhibiting gastric motility, and to a major extent, in all gastric secretion (37). Regulation of ghrelin secretion by clonidine or other molecules with respect to GH status suggests specific actions of some molecules, as observed on dexamethasone administration, and needs further evaluation (28).

The role of the GH status with respect to ghrelin secretion is still a matter of debate. Janssen et al. reported that GHD adults had similar ghrelin levels as those seen in controls and that a 1-year GH replacement therapy failed to modulate circulating ghrelin levels. The lack of modulation of ghrelin secretion with respect to baseline levels after a relatively prolonged GH therapy was hypothesized to be a consequence of a balance between reduction of adiposity and insulin resistance and increased circulating GH levels (38). Similarly, in some studies, ghrelin levels were similar in GHD and GHS children and adults at fasting (28, 39-43). These findings are consistent with our results that suggest that ghrelin does not modulate the quantity but mainly quality of GH secretion by modulating the GH pulse amplitude (40). In contrast, Giavioli et al. showed lower ghrelin levels in GHD adults, which increased after a long-term treatment with recombinant human growth hormone (rhGH) possibly because of modifications of body fat stores rather than of GH–IGF-I levels (40). These findings are also in contrast with those of Engstrom who recorded a decrease in total ghrelin levels after 9 months of rhGH treatment in GHD subjects (42). These discordant data may be attributed to patients’ selection, group size, dose of rhGH treatments, and other comorbidities and need to be investigated further.

Similarly, in our study, AG levels were not modulated by ARG infusion. To our knowledge, this is the first study that aimed to evaluate AG secretion with respect to amino acid regulation. A recent study failed to show modulation of AG with respect to GH status in humans (44).

In conclusion, this study shows that there is no correlation between GH and total ghrelin levels and AG levels in prepubertal GHD and GHS children at fasting or during a classical GH-provocative test. ARG infusion could not blunt ghrelin secretion irrespective of the GH status in childhood. Moreover, since ARG influences GH secretion via modulation of SRIH release, ghrelin secretion seems to be partially refractory to SRIH action.
